# Genome-Based Discovery of a Novel Membrane-Bound 1,6-Dihydroxyphenazine Prenyltransferase from a Marine Actinomycete

**DOI:** 10.1371/journal.pone.0099122

**Published:** 2014-06-03

**Authors:** Philipp Zeyhle, Judith S. Bauer, Jörn Kalinowski, Kazuo Shin-ya, Harald Gross, Lutz Heide

**Affiliations:** 1 Pharmazeutische Biologie, Pharmazeutisches Institut, Eberhard Karls Universität Tübingen, Tübingen, Germany; 2 Microbial Genomics and Biotechnology, Center for Biotechnology, Bielefeld University, Bielefeld, Germany; 3 Biomedical Research Institute, National Institute of Advanced Industrial Science and Technology (AIST), Tokyo, Japan; University of New South Wales, Australia

## Abstract

Recently, novel prenylated derivatives of 1,6-dihydroxyphenazine have been isolated from the marine sponge-associated *Streptomyces* sp. SpC080624SC-11. Genome sequencing of this strain now revealed a gene cluster containing all genes necessary for the synthesis of the phenazine and the isoprenoid moieties. Unexpectedly, however, the cluster did not contain a gene with similarity to previously investigated phenazine prenyltransferases, but instead a gene with modest similarity to the membrane-bound prenyltransferases of ubiquinone and menaquinone biosynthesis. Expression of this gene in *E. coli* and isolation of the membrane fraction proved that the encoded enzyme, Mpz10, catalyzes two successive prenylations of 1,6-dihydroxyphenazine. Mpz10 is the first example of a membrane-bound enzyme catalyzing the prenylation of a phenazine substrate, and one of few examples of membrane-bound enzymes involved in the prenylation of aromatic secondary metabolites in microorganisms.

## Introduction

**Figure 1:**
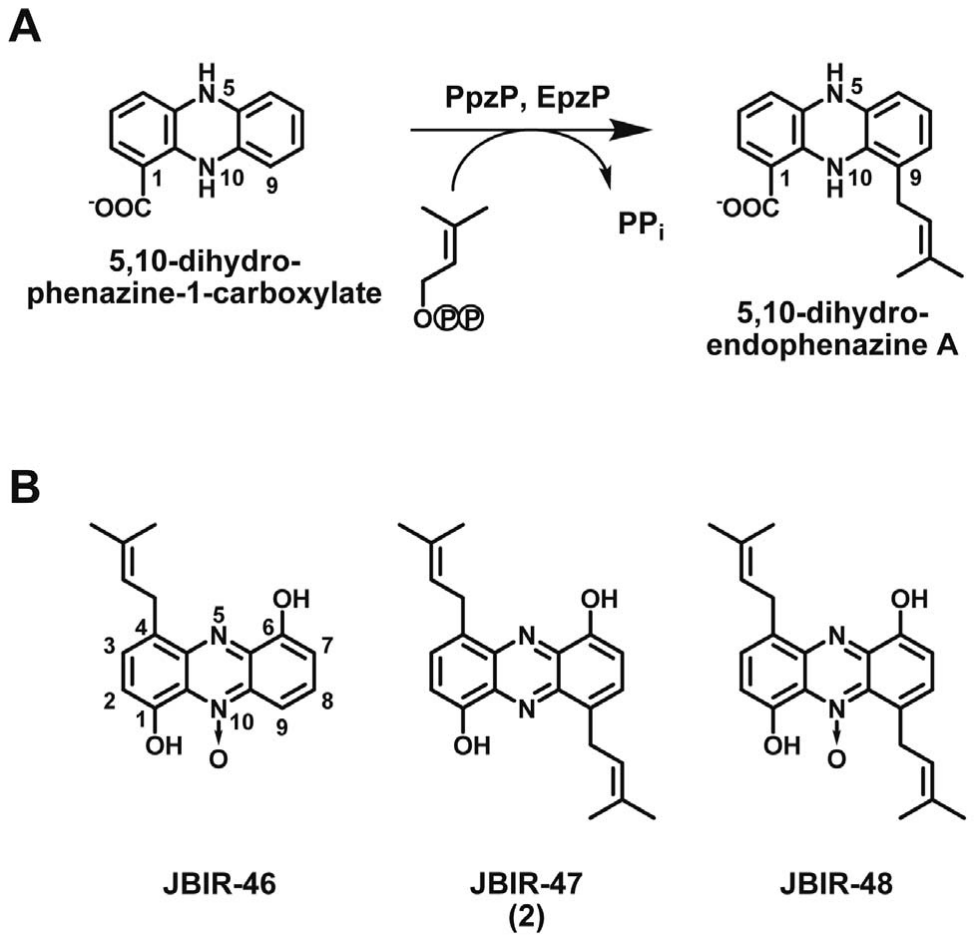
Prenylation in the biosynthesis of phenazines. (A) Reaction catalyzed by the previously discovered prenyltransferases PpzP and EpzP. (B) Structures of the phenazines JBIR-46, -47, and -48 from *Streptomyces* sp. SpC080624SC-11.

Phenazines are important microbial secondary metabolites. They are formed by actinobacteria, by certain groups of Gram-negative proteobacteria (especially *Pseudomonas* strains), and by few archaea [1]. Phenazines show promising antibacterial, antifungal, antitumor, and neuronal cell-protecting activities, and they act as virulence factors in pathogenesis [2]. Besides their role as antibiotics [3], they have various functions for the producing cell, often related to their capability to shuttle electrons by reversible oxidation and reduction [4]. *Pseudomonas* strains produce mostly simple phenazines such as phenazine-1-carboxylic acid (PCA), and the biosynthesis of these compounds has been studied in detail [1]. In actinobacteria a much greater structural diversity of phenazines can be found, including many isoprenylated phenazines [2]. However, only four gene clusters for phenazine biosynthesis have been investigated in actinobacteria, including two which direct the biosynthesis of so-called endophenazines, i.e. mono-isoprenylated phenazines [5]–[7]. A central reaction in the biosynthesis of endophenazines is the *C*-prenylation of the reduced form of PCA under catalysis of PpzP [5] or of the very similar EpzP (Figure 1A). The X-ray structure of EpzP [8] proved that it belongs to the ABBA prenyltransferases, a recently identified and now rapidly expanding class of enzymes involved in the biosynthesis of a diverse range of prenylated aromatic secondary metabolites in bacteria and fungi [9], [10]. The soluble, stable, and mostly Mg^2+^-independent ABBA prenyltransferases are of considerable interest for chemoenzymatic synthesis, motivating an ongoing search for new members of this class with new substrate specificities [11], [12].

From the marine sponge-associated *Streptomyces* strain SpC080624SC-11, recently the bis-isoprenylated phenazines JBIR-47 and JBIR-48 have been isolated, together with the mono-isoprenylated JBIR-46 [13], [14]. The structure of these compounds (Figure 1B) suggests that the prenyltransferase involved in their biosynthesis should have a different substrate specificity than the previously examined enzymes PpzP and EpzP. Using a genome-based approach, we therefore attempted to identify and characterize the responsible enzyme. Unexpectedly, we found that this prenyltransferase is unrelated to the soluble enzymes PpzP and EpzP, and represents a rather unique integral membrane protein, distantly related to the prenyltransferase UbiA of ubiquinone biosynthesis. This shows that nature has devised two completely different types of biocatalysts for prenylated phenazine biosynthesis in *Streptomyces*. Our study adds another example to the very small number of membrane-bound aromatic prenyltransferases involved in microbial secondary metabolism.

## Results

### Analysis of the genome sequence of *Streptomyces* sp. SpC080624SC-11

**Figure 2:**
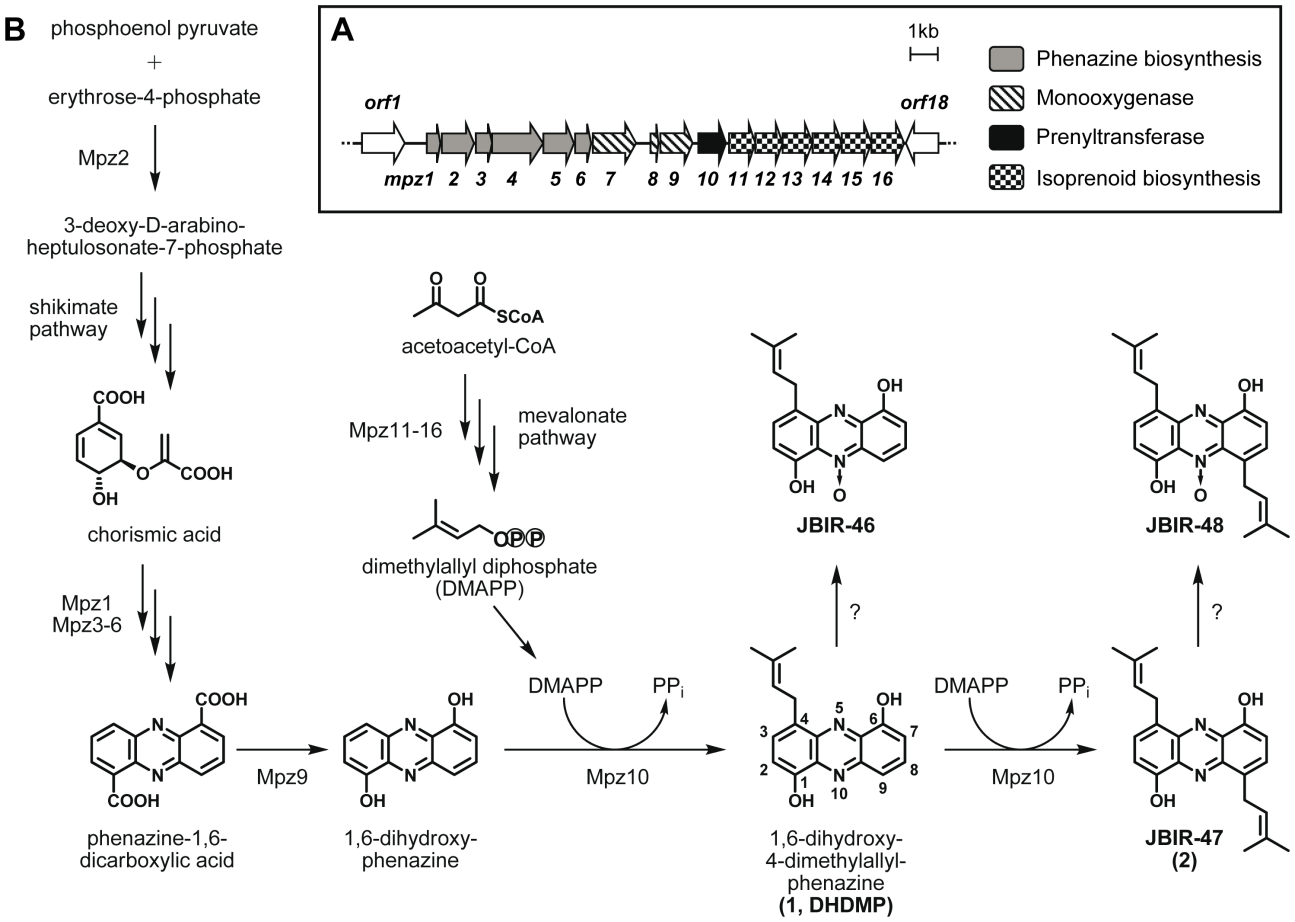
Phenazine biosynthesis in *Streptomyces* sp. SpC080624SC-11. (A) Putative biosynthetic gene cluster of JBIR-46, -47, and -48. (B) Proposed pathway for the biosynthesis of JBIR-46, -47, and -48. See Table 1 for additional information on the genes contained in the depicted gene cluster.

Sequencing of the genomic DNA of *Streptomyces* sp. SpC080624SC-11 and the assembly of the sequence reads led to a draft genome sequence. A local BLASTP search for phenazine biosynthesis and mevalonate pathway genes readily identified a putative gene cluster for the biosynthesis of the prenylated phenazines JBIR-46, -47, and -48. The cluster spans 17.8 kb and comprises 16 putative coding sequences (Figure 2A, Table 1). Six of the genes, designated as *mpz1-6*, show high similarity (81-61% at the amino acid level) to the phenazine biosynthesis genes *phzBCDEFG* from *Pseudomonas fluorescens* [15].

**Table 1:** Genes in the putative biosynthetic gene cluster for JBIR-46, -47, and -48.

Gene	Size (bp/aa)	Proposed Function	Orthologue identified by BLASTP search	Identity/Similarity [%]
*orf1*	1638/545	ATPase	SZN_31494, *Streptomyces zinciresistens*	85/89
*mpz1*	486/161	Phenazine biosynthesis protein	PhzB, *Pseudomonas fluorescens*	69/81
*mpz2*	1224/407	3-Deoxy-D-arabino-heptulosonate 7-phosphate synthase	PhzC, *Pseudomonas fluorescens*	49/61
*mpz3*	573/190	2,3-Dihydro-3-hydroxy-anthranilate synthase	PhzD, *Pseudomonas fluorescens*	56/73
*mpz4*	1941/646	2-Amino-2-desoxy-isochorismate synthase	PhzE, *Pseudomonas fluorescens*	54/69
*mpz5*	1167/388	*trans*-2,3-Dihydro-3-hydroxyanthranilate isomerase	PhzF, *Pseudomonas fluorescens*	60/70
*mpz6*	636/211	FMN-dependent oxidase	PhzG, *Pseudomonas fluorescens*	48/65
*mpz7*	1623/540	Monooxygenase	ChnB, *Brachymonas petroleovorans*	56/74
*mpz8*	330/109	Monooxygenase	TcmH, *Streptomyces glaucescens*	48/60
*mpz9*	1215/404	Flavin dependent hydroxylase	PhzS, *Pseudomonas aeruginosa*	51/62
*mpz10*	996/331	Prenyltransferase	O3I_007965, *Nocardia brasiliensis* ATCC 700358	33/51
*mpz11*	1020/339	Mevalonate kinase	MK, *Streptomyces cinnamonensis*	57/71
*mpz12*	1044/347	Mevalonate diphosphate decarboxylase	Mcl8, *Streptomyces* sp. CNH189	73/80
*mpz13*	1140/379	Phosphomevalonate kinase	PMK, *Streptomyces* sp. KO-3988	57/70
*mpz14*	1092/363	Isopentenyl diphosphate isomerase	Mcl6, *Streptomyces* sp. CNH189	74/86
*mpz15*	1068/355	3-Hydroxy-3-methylglutaryl CoA reductase	HMGR, *Streptomyces anulatus*	80/89
*mpz16*	1185/394	3-Hydroxy-3-methylglutaryl CoA synthase	Mcl4, *Streptomyces* sp. CNH189	76/87
*orf18*	1212/403	integrase catalytic subunit	OCO_06920, *Mycobacterium intracellulare* MOTT-02	48/63

The six genes *mpz11-16* show obvious similarity (89-70%) to genes coding for enzymes of the mevalonate pathway, corresponding to the fact that the isoprenoid moieties of JBIR-46, -47, and -48 originate from the mevalonate pathway [14].

Four genes are situated between the phenazine and mevalonate genes (Figure 2A) and may be involved in the tailoring of the phenazine scaffold. Mpz9 is similar (62%) to PhzS from *Pseudomonas aeruginosa*. PhzS has been shown to catalyze the decarboxylative hydroxylation of PCA to 1-hydroxyphenazine [16], and Mpz9 may carry out a similar reaction in JBIR-46, -47, and -48 biosynthesis (Figure 2B). *mpz7* and *mpz8* show similarity (74% and 60%, respectively) to monooxygenases. It is tempting to speculate that they may be involved in the formation of the *N*-oxide group found in JBIR-46 and JBIR-48, but so far there is no direct evidence to support this speculation. The closest functionally characterized orthologues to these genes are *chnB* from *Brachymonas petroleovorans* and *tcmH* from *Streptomyces glaucescens*, respectively [17], [18]. These genes code for monooxygenases which oxidize alicyclic or aromatic compounds, but are not involved in *N*-oxidation reactions.

Contrary to our expectations, no gene with similarity to the phenazine prenyltransferases PpzP/EpzP or other ABBA prenyltransferase genes could be identified in the cluster, nor in the entire genome of this strain. However, we noticed that *mpz10* codes for a protein with moderate similarity (51%) to putative 4-hydroxybenzoate polyprenyltransferases.

The predicted gene product of *mpz10* is an enzyme of 331 amino acids with a calculated mass of 36.3 kDa. It contains eight transmembrane helices as predicted by the TMHMM Server (v. 2.0) [19]. Sequence comparison revealed only moderate similarity to previously characterized enzymes, e.g. 19.6% identity to UbiA and 18.6% to MenA, the membrane-bound prenyltransferases of ubiquinone and menaquinone biosynthesis in *E. coli*. This raised the question whether Mpz10 may catalyze the prenylation of phenazines in the biosynthesis of JBIR-46, -47, and -48.

### Generation of 1,6-dihydroxyphenazine as a substrate for Mpz10

We speculated that 1,6-dihydroxyphenazine may be the substrate of the first prenylation reaction in the biosynthesis of JBIR-46, -47, and -48 (Figure 2B). In order to test whether Mpz10 may catalyze this reaction, we generated 1,6-dihydroxyphenazine, using a modification of a previously published approach [20]. A culture of the actinomycete *Brevibacterium iodinum*, which is known to produce iodinin (1,6-dihydroxyphenazine-5,10-dioxide), was extracted with dichloromethane. This extract was reduced with hydrogen under platinum catalysis and the resulting product was purified over a silica gel column to give 1,6-dihydroxyphenazine (yield: 31.6 mg from 1.25 l culture). The structure of 1,6-dihydroxyphenazine was confirmed via LC-MS, ^1^H, and ^13^C NMR.

### Expression of Mpz10 and confirmation of its prenyltransferase activity

**Figure 3:**
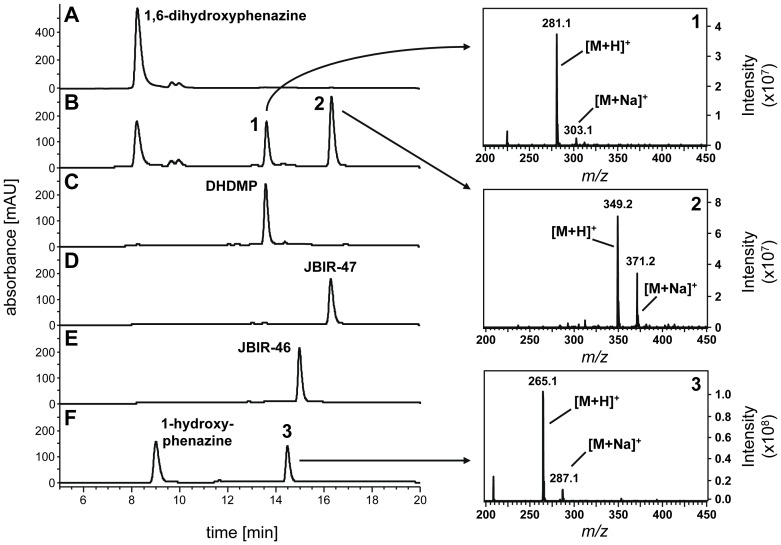
HPLC and LC-MS analysis of the reaction products of Mpz10. (A) and (B): Incubations containing 1,6-dihydroxyphenazine, dimethylallyl diphosphate (DMAPP), and Mg^2+^ with the membrane fractions of *E. coli* harboring either the empty vector pET-28a(+) (A) or the Mpz10 expression vector pPH23 (B). (C) Authentic DHDMP (structure see Figure 2B). (D) and (E): Authentic JBIR-47 and JBIR-46 (structures see Figure 1B). (F) Incubation containing 1-hydroxyphenazine, DMAPP, and Mg^2+^ with the membrane fraction of *E. coli* harboring the Mpz10 expression vector pPH23 (for the structure of **3** see Figure 5). Mass spectra of the three enzymatic products are shown on the right. Detection: A-E, UV 275 nm; F, UV 368 nm.

For expression in *E. coli* the gene *mpz10* was amplified from genomic DNA of *Streptomyces* sp. SpC080624SC-11 and cloned in the vector pET-28a(+), resulting in the expression construct pPH23. pPH23 was transformed into *E. coli* and Mpz10 was expressed using induction with IPTG (see Experimental Procedures). Enzyme extracts were generated and incubated with 1,6-dihydroxyphenazine and dimethylallyl diphosphate (DMAPP) in the presence of Mg^2+^. This resulted in the rapid formation of the two products **1** and **2** (Figure 3B). LC-MS analysis showed [M+H]^+^ ions at *m/z* 281 and *m/z* 349 for **1** and **2**, respectively, which suggested that these compounds represent a monoprenylated and a diprenylated derivative of 1,6-dihydroxyphenazine. Incubation with extracts from *E. coli* cells harboring only the empty vector pET-28a(+) yielded no product formation (Figure 3A). This suggested that Mpz10 catalyzes the transfer of two dimethylallyl moieties onto 1,6-dihydroxyphenazine.

### Localization of Mpz10

To confirm the localization of Mpz10 in the membrane, a crude protein extract was subjected to centrifugation at 100,000×*g*. The crude extract, the membrane fraction, and the supernatant of the ultracentrifugation were assayed for prenyltransferase activity (Table 2). Nearly all of the activity originally found in the crude protein extract was recovered in the membrane fraction after ultracentrifugation, with only negligible activity remaining in the supernatant. Isolation of the membrane fraction by ultracentrifugation resulted in a 2.3-fold increase of activity in the membrane fraction, clearly demonstrating the localization of the prenyltransferase activity in the membrane of *E. coli*. All further biochemical investigations were carried out with the membrane fraction.

**Table 2:** Localization of the Mpz10 activity in the membrane fraction.

Fraction	Total protein	Product formation		Specific activity	
	[mg]	[nmol s^−1^]	[%]	[pmol s^−1^ mg^−1^]	[%]
Crude protein extract	891.9	83.4	100.0	93.5	100.0
Supernatant (100,000×*g*)	579.8	0.7	0.8	1.2	1.3
Membrane fraction	301.8	65.6	78.7	217.5	232.6

Comparison of the prenyltransferase activity of different cell fractions. Activity was tested with 1,6-dihydroxyphenazine, DMAPP, and Mg^2+^ as described in the Experimental Procedures. The membrane fraction was obtained by centrifugation at 100,000×*g*.

### Identification of the products of the Mpz10 reaction

**Figure 4:**
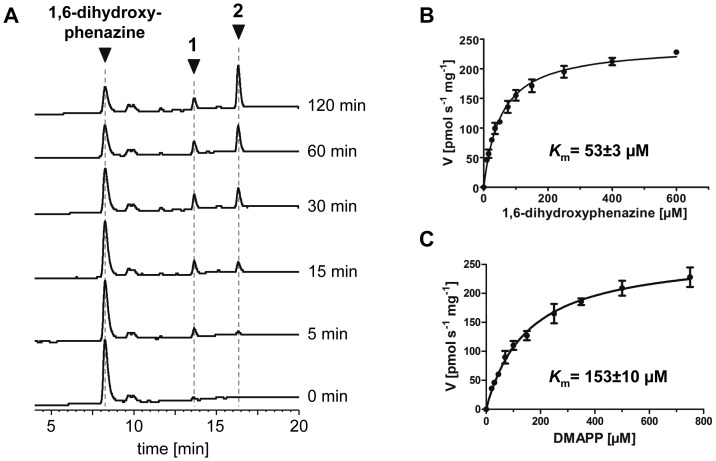
Biochemical investigation of the Mpz10 reaction. (A) HPLC analysis of the time-dependent formation of products **1** and **2**. Detection: UV, 275 nm. (B) and (C): Product formation at different concentrations of 1,6-dihydroxyphenazine and dimethylallyl diphosphate (DMAPP). In the experiments shown in (B), DMAPP was kept constant at 0.5 mM. In the experiments shown in (C), 1,6-dihydroxyphenazine was kept constant at 0.2 mM. *K*_m_ values were determined by nonlinear regression, using GraphPad Prism software. Data represent mean ± SD of triplicate determinations.

When the membrane fraction containing Mpz10 was incubated with 1,6-dihydroxyphenazine, DMAPP, and Mg^2+^, HPLC analysis showed formation first of product **1**. After 30 min and longer, **2** became the dominant product, while the amount of **1** decreased (Figure 4A). As mentioned above, LC-MS analysis showed that **1** and **2** are mono- and diprenylated derivatives of 1,6-dihydroxyphenazine, respectively. The enzymatic products were compared with authentic reference samples of 1,6-dihydroxy-4-dimethylallyl-phenazine (DHDMP) and 1,6-dihydroxy-4,9-bis-(dimethylallyl)-phenazine, i.e. JBIR-47 (Figure 2B). JBIR-47 had been isolated and structurally elucidated previously, together with JBIR-46 and JBIR-48 [14]. To gain a sample of DHDMP, an authentic standard of JBIR-46 (Figure 3E) was reduced to DHDMP (Figure 3C) with sodium dithionite. The successful reduction of JBIR-46 was confirmed via LC-MS. Comparison of the enzymatic products **1** and **2** with the samples of DHDMP and JBIR-47 by LC-MS/MS revealed that product **1** has the same retention time, UV spectrum, molecular ion, and fragmentation pattern as DHDMP (Figure 3B and 3C). Likewise, **2** has the same retention time, UV spectrum, molecular ion, and fragmentation pattern as JBIR-47 (Figure 3B and 3D). Therefore, Mpz10 catalyzes the two successive prenylation reactions expected to occur in the biosynthesis of JBIR-47 and JBIR-48 (Figure 2B).

### Biochemical properties of Mpz10

In the assay described under Experimental Procedures, product formation showed linear dependence on the amount of membrane protein (up to 0.5 mg ml^-1^) and on the reaction time (up to 20 min). Mpz10 activity was strictly dependent on the presence of Mg^2+^ or other divalent cations. The optimal concentration of Mg^2+^ ions was between 2 and 10 mM. Substitution of Mg^2+^ by Fe^2+^ or Zn^2+^ (10 mM) reduced the activity to 13.5% or 11.8% of the value found in presence of Mg^2+^, respectively. Only traces of products were detected in the presence of Co^2+^, Mn^2+^, or Ni^2+^, and no product formation was observed in the presence of Ca^2+^ or Cu^2+^. Addition of 2 mM EDTA instead of metal ions completely abolished the product formation. Like farnesyl diphosphate synthase and the membrane-bound prenyltransferases of lipoquinone biosynthesis [11], [21], Mpz10 contains two aspartate-rich motifs, i.e. NALADRWED (AA 89-97) and DYFDD (AA 220-224). These are expected to bind the isoprenoid diphosphate substrate in form of its complex with a Mg^2+^ ion [22], [23], consistent with the observed absolute requirement of Mpz10 for divalent cations.

Product formation in the assay was also strictly dependent on the presence of membrane fraction harboring Mpz10, of 1,6-dihydroxyphenazine, and of DMAPP. The enzyme showed highest activity at a pH of 8.1, with half maximal values at pH 5.5 and 9.0. The addition of NaCl (100 mM) had no influence on enzyme activity. The Mpz10 reaction displayed Michaelis-Menten kinetics. The apparent *K*_m_ values for 1,6-dihydroxyphenazine and for DMAPP were determined at 53 µM and 153 µM, respectively (Figure 4B and 4C). The maximal velocity of the reaction was 255 pmol s^-1^ (mg protein)^-1^.

### Substrate specificity of Mpz10

Mpz10 was found to possess narrow specificity for its aromatic substrate and strict specificity for DMAPP as its isoprenoid substrate. When geranyl diphosphate or farnesyl diphosphate were used instead of DMAPP in incubations with 1,6-dihydroxyphenazine, no product formation was observed. When naringenin, a genuine substrate of prenylflavonoid biosynthesis in plants [24] was used as aromatic substrate, no product formation could be observed with any of the isoprenoid substrates.

Mpz10 was also incubated with different phenolic compounds previously identified as substrates of ABBA prenyltransferases (1,6-dihydroxynaphthalene and flaviolin, i.e. 2,5,7-trihydroxynaphthoquinone). Of these, only flaviolin was prenylated in the presence of DMAPP. LC-MS showed a peak at *m/*z 273 [M-H]^-^, corresponding to a monoprenylated flaviolin derivative. However, the reaction velocity was very low, preventing a precise quantification. Notably, 5,10-dihydrophenazine-1-carboxylate, the genuine substrate of the phenazine prenyltransferases PpzP and EpzP, was not accepted by Mpz10, and neither was phenazine-1-carboxylate. However, incubation of Mpz10 with 1-hydroxyphenazine and DMAPP led to the formation of product **3** (Figure 3F). In LC-MS analysis **3** showed a molecular ion at *m/z* 265 [M+H]^+^, which suggested it to be a monoprenylated derivative of 1-hydroxyphenazine. Therefore, a preparative scale assay was carried out, and the product was isolated and purified (see Experimental Procedures).

**Figure 5:**
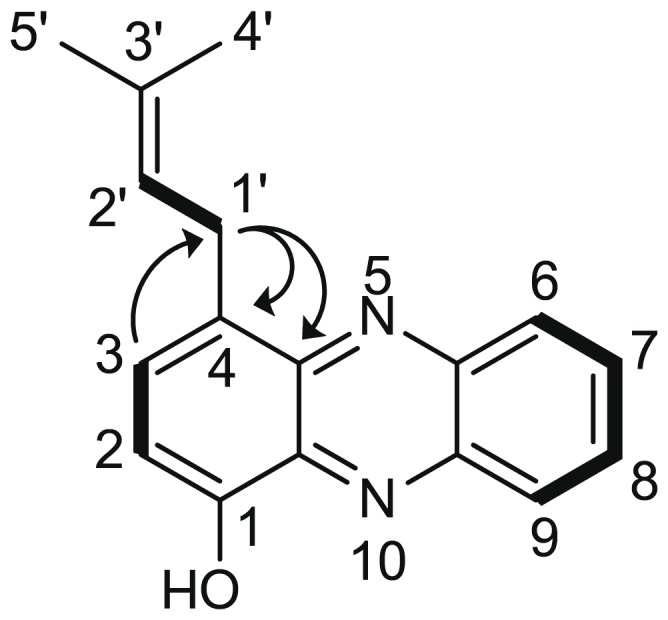
Structure of the enzymatic product 1-hydroxy-4-dimethylallyl-phenazine (3). Bold lines indicate key ^1^H-^1^H COSY and arrows key ^1^H-^13^C HMBC correlations.

NMR spectroscopic investigations in comparison to the educt 1-hydroxyphenazine confirmed that product **3** was 1-hydroxy-4-dimethylallyl-phenazine (Figure 5). The ^13^C NMR spectrum of **3** revealed five additional carbon resonances (Table 3, Figure S1), which were attributable to a dimethylallyl moiety. In the ^1^H NMR spectrum of **3**, the absence of the H-4 resonance signal at *δ* 7.73 and a high-field shift of the methine group H-3 along with the collapse of its coupling pattern from a doublet of doublet (dd) system to a doublet (d) was observed (Table 3, Figure S2). This provided proof that the prenylation had occurred at carbon C-4, in *para* position to the hydroxyl group. These findings were further corroborated by 2D NMR experiments, particularly by ^1^H-^13^C HMBC cross correlations between methylene H_2_-1′ and carbons C-3, C-4, and C-4a of the phenazine skeleton (Figure 5). The apparent *K*_m_ of 1-hydroxyphenazine was determined to be 9.8 µM and the maximal velocity of the reaction to be 59.9 pmol s^-1^ (mg protein)^-1^.

**Table 3:** NMR spectroscopic data for 1-hydroxyphenazine and the enzymatic product 3 (structure see Figure 5).

	1-hydroxyphenazine		3		
position	*δ*_H_ a ^,b^	*δ*_C_^b,c,d^	*δ*_H_^e^	*δ*_C_^d,f^	HMBC^g^
1		153.7 qC		151.7 qC	
2	7.23 (dd, *J* = 7.4, 1.2)	110.1 CH	7.15 (d, *J* = 7.6)	109.9 CH	1, 4, 10a
3	7.83 (dd, *J* = 8.9, 7.4)	132.7 CH	7.59 (d, *J* = 7.6)	130.4 CH	1, 4a, 1′
4	7.73 (dd, *J* = 8.9, 1.2)	120.5 CH		131.5 qC	
4a		144.8 qC		143.3 qC	
5a		144.9^j^ qC		143.8^j^ qC	
6	8.22^h^(m)	130.5^h^ CH	8.26^h^ (d, *J* = 8.4)	130.7^h^ CH	8, 9a
7	7.93^i^ (m)	131.4^i^ CH	7.92^i^ (m)	131.4^i^ CH	5a, 9
8	7.93^i^ (m)	131.7^i^ CH	7.92^i^ (m)	131.3^i^ CH	6, 9a
9	8.22^h^ (m)	130.1^h^ CH	8.20^h^ (d, *J* = 8.4)	129.9^h^ CH	5a, 7
9a		142.1^j^ qC		141.7^j^ qC	
10a		136.0 qC		136.2 qC	
1′			3.98 (d, *J* = 7.4)	29.4 CH_2_	4, 4a, 2′, 3′
2′			5.50 (t, *J* = 7.4)	124.0 CH	1′, 4′
3′				132.9 qC	
4′			1.84 (s)	18.0 CH_3_	2′, 3′, 5′
5′			1.72 (s)	25.9 CH_3_	2′, 3′, 4′
OH	9.23 (br)		9.01 (br)		

NMR spectra were recorded in acetone-*d*_6_ (*δ* in ppm, *J* in Hz). The corresponding ^13^C and ^1^H NMR spectra are depicted in Figure S1 and Figure S2, respectively.

a Recorded at 400 MHz. ^b^Measured values were in good agreement with published NMR data for 1-hydroxy- or 1-methoxy-phenazine [20], [52]. ^c^Recorded at 101 MHz. ^d^Multiplicity determined by a multiplicity edited ^1^H-^13^C HSQC NMR experiment. ^e^Recorded at 600 MHz. ^f^Recorded at 151 MHz. ^g^Protons showing long-range correlation with indicated carbon. ^h,i,j^Assignments interchangeable.

When 4-hydroxybenzoate, the genuine substrate of the membrane-bound prenyltransferase UbiA of ubiquinone biosynthesis [25], was incubated with geranyl diphosphate or farnesyl diphosphate, a low product formation was observed. However, these prenylated products appeared also in incubations with the membrane fraction from strains harboring only the empty vector pET-28a(+) and therefore are most likely formed under catalysis of the UbiA homologue of the expression host *E. coli* Rosetta2(DE3)pLysS.

## Discussion

In the marine sponge-associated bacterial strain *Streptomyces* sp. SpC080624SC-11, a new class of prenylated phenazines has been discovered and the respective compounds were termed JBIR-46, JBIR-47, and JBIR-48 (Figure 1B) [14]. Using a genome-based approach, we now identified a putative gene cluster for the biosynthesis of these compounds. The cluster contains a unique prenyltransferase which catalyzes two successive prenylations of 1,6-dihydroxyphenazine resulting in the formation of JBIR-47.

The genetic and biochemical data revealed in our study allow the formulation of a hypothetical pathway to the prenylated phenazines as depicted in Figure 2B. The isoprenoid moieties are formed via the mevalonate pathway [14], like the isoprenoid moieties of the previously investigated endophenazines [26]. In the presently investigated strain, the six genes from *mpz11* to *mpz16* code for all enzymes required for the pathway leading from acetoacetyl-CoA to DMAPP. Notably, the previously identified endophenazine biosynthetic gene clusters possess an additional mevalonate pathway gene, coding for a unique microbial acetoacetyl-CoA synthase which catalyzes the condensation of acetyl-CoA and malonyl-CoA [27], [28]. However, this acetoacetyl-CoA synthase is not essential for isoprenoid biosynthesis via mevalonate, since also another pathway exists for acetoacetyl-CoA formation [27].

The six gene operon from *mpz1* to *mpz6* is similar to *phzBCDEFG* from *Pseudomonas fluorescens* and is likely to direct the biosynthesis of the phenazine skeleton from precursors of the shikimate pathway [29]. However, the presently investigated strain lacks an orthologue of *phzA* which in *Pseudomonas* forms an operon with *phzBCDEFG*. *phzA* is similar in size and sequence to *phzB*, and the gene products of both are likely to be involved in the linkage of two chorismate-derived precursors which gives rise to the tricyclic ring system of the phenazines [30]. It has been suggested [1] that organisms which contain orthologues of both *phzA* and *phzB*, such as the producer strains of endophenazines [5], [6], produce only PCA as key intermediate in the biosynthesis of substituted phenazines. In contrast, organisms which lack an orthologue of *phzA* have been suggested to produce phenazine-1,6-dicarboxylic acid (besides some PCA) as key intermediate, leading to differently substituted phenazines. This hypothesis appears to be consistent with the genes and compounds found in the presently investigated *S*. sp. SpC080624SC-11 (Figure 2B): phenazine-1,6-dicarboxylic acid is expected to undergo two decarboxylative hydroxylations under catalysis of Mpz9, in analogy to the reaction catalyzed by the similar PhzS of *Pseudomonas aeruginosa* [31], yielding 1,6-dihydroxyphenazine. If PCA would be used by Mpz9 in the same reaction, the product would be 1-hydroxyphenazine. Interestingly, both 1-hydroxyphenazine and 1,6-dihydroxyphenazine were accepted by the prenyltransferase Mpz10 with similar catalytic efficiency. Prenylated derivatives of 1,6-dihydroxyphenazine have been identified in this strain previously [14], but based of the substrate tolerance of Mpz10 also derivatives of 1-hydroxyphenazine may be expected to occur.

A principal surprise in the present study was that the prenyltransferase involved in the biosynthesis of JBIR-46, -47, and -48 was completely unrelated to the phenazine prenyltransferases PpzP and EpzP which had previously been identified in other streptomycetes. So far, the *C*-prenylation of aromatic compounds in the very diverse secondary metabolism of actinomycetes has been found to be catalyzed by soluble enzymes of the so-called ABBA superfamily [9], [10]. The fungal indole prenyltransferases, involved e.g. in ergot alkaloid biosynthesis, were found also to belong to this superfamily [9], [32]. In contrast, membrane-bound prenyltransferases with aromatic substrates in bacteria remained restricted to primary metabolism, i.e. to the biosynthesis of ubiquinones and menaquinones [11]. A membrane-bound aromatic prenyltransferase involved in microbial secondary metabolism was functionally identified for the first time in 2010 by in vivo studies on a fungal meroterpenoid gene cluster [33]. Subsequently, three additional membrane-bound prenyltransferases of fungal meroterpenoid biosynthesis were identified by in vivo studies and bioinformatic sequence analysis [34]–[36]. In bacteria, a membrane-bound farnesyltransferase from a myxobacterium catalyzing the *C*-prenylation of a hydroxyquinoline [37], and a prenyltransferase catalyzing the *O*-prenylation of a small polyketide in a rare *Actinomyces* strain [38] were investigated in vitro. Bioinformatic sequence analyses predict the existence of further membrane-bound prenyltransferases in bacterial secondary metabolism, e.g. in the biosynthesis of BE-40644 in an *Actinoplanes* strain [39] and in aurachin RE biosynthesis in *Rhodococcus erythropolis* [40]. All these enzymes are integral transmembrane proteins with similarity to the prenyltransferases of menaquinone and ubiquinone biosynthesis [41], [42]. They contain typical aspartate-rich motifs (e.g. NxxxDxxxD) for binding of the isoprenoid substrate via a Mg^2+^ ion [11]. There is no experimentally determined structure for these transmembrane enzymes, but a structure model has been proposed for UbiA [22]. Membrane-bound aromatic prenyltransferases have also been identified in plant secondary metabolism [43].

Using a cluster analysis as described previously [9], Mpz10 identified in the present study did not group with prenyltransferases of the UbiA or MenA type, nor with any other group of previously reported enzymes. The sequence identity of Mpz10 (comprising 331 amino acids) to UbiA (290 aa) and MenA (308 aa) is low (19.6% and 18.6%, respectively), making it a rather unique enzyme.

The discovery of Mpz10, as well as the mentioned cluster analysis of membrane-bound prenyltransferases in bacterial genomes [9] indicates that membrane-bound prenyltransferases may be involved much more frequently in bacterial secondary metabolism than recognized previously. Notably, the genome sequence of SpC080624SC-11 contains two further genes annotated as “4-hydroxybenzoate polyprenyltransferase” and “UbiA prenyltransferase”, respectively. Streptomycetes are Gram-positive organisms, assumed to produce menaquinones but not ubiquinones [44]. Since no homologue of MenA of *E. coli* was found in the genome of SpC080624SC-11, one of the above-mentioned two genes may be involved in menaquinone biosynthesis, but the function of the other one remains unknown. As a further example, the genome of *Streptomyces violaceusniger* Tü 4113 contains one gene annotated as “4-hydroxybenzoate polyprenyltransferase” (YP_004810797) and two genes annotated as “UbiA prenyltransferase” (YP_004812627 and YP_004814952). It is tempting to speculate that some of these automatically annotated “UbiA-like polyprenyltransferases” may be involved in the biosynthesis of so far unknown secondary metabolites. Many different prenylated phenazines have been reported to occur in bacteria [2], [45] and it remains to be determined whether the responsible prenyltransferases belong to the soluble ABBA prenyltransferase superfamily, or whether they are membrane-bound enzymes like Mpz10 discovered in the present study.

## Experimental Procedures

### Chemicals

Sea salts and (±)-naringenin were purchased from Sigma-Aldrich (Steinheim, Germany). 4-hydroxybenzoic acid and 1,6-dihydroxynaphthalene were obtained from Acros Organics (New Jersey, USA). 1-hydroxyphenazine was from TCI (Zwijndrecht, Belgium), kanamycin was from Roth (Karlsruhe, Germany), and chloramphenicol was from Genaxxon BioScience (Biberach, Germany). Diaion HP-20 was received from Supelco (Bellefonte, USA) and PCA from InFarmatik (Hungary). Acetone-*d_6_* was purchased from Deutero (Kastellaun, Germany). Reference samples of JBIR-46 and -47 were isolated as described previously [14]. Flaviolin was isolated as described by Gross et al. [46] and DMAPP, geranyl diphosphate, and farnesyl diphosphate were synthesized as described previously [47]. Restriction enzymes were bought from New England Biolabs (Frankfurt am Main, Germany).

### Bacterial strains and culture conditions

*Streptomyces* sp. SpC080624SC-11 has been isolated previously from a marine sponge [13]. It was grown in liquid or on solid ISP medium 2 [48] containing 20 g l^−1^ sea salts at 27°C. *Brevibacterium iodinum* DSM 433 was obtained from the Deutsche Sammlung von Mikroorganismen und Zellkulturen GmbH (Braunschweig, Germany) and was cultured in liquid or on solid DSMZ medium 1 at 30°C. *Escherichia coli* XL1 Blue MRF′ (Stratagene) was used for cloning and was grown in liquid or on solid Luria-Bertani medium at 37°C. Kanamycin (50 µg ml^−1^) and chloramphenicol (25 µg ml^−1^) were used to select recombinant strains.

### General analytical procedures

HPLC analysis was carried out using an Agilent 1100 series system coupled with a photodiode array detector. UV and FT-IR spectra were obtained employing a Perkin Elmer Lambda25 and a Jasco FT/IR-4200 instrument, respectively. All NMR spectra were recorded on Bruker Avance III 400 and 600 spectrometers. Spectra were referenced to the residual solvent signal of acetone-*d*_6_ with resonances at *δ*_H/C_ 2.04/29.8. HR-ESI-TOF-MS data were recorded on a Bruker maXis 4G mass spectrometer. ESI-LC/MS experiments were carried out using an Agilent 1200 series system coupled with an ESI spectrometer (LC/MSD Ultra Trap System XCT 6330).

### Genetic procedures and genome sequencing

Standard techniques for DNA isolation and manipulation were used according to Kieser et al. [49] and Sambrook and Russell [50]. PCR fragments were isolated from agarose gels by using Justspin columns (Genaxxon BioScience). Genomic DNA was purified over Genomic-tip 100/G columns (Qiagen). The preparation of an 8kPE and a WGS library was performed according to standard protocols from Roche Applied Science. The Genome Sequencer FLX System and Titanium chemistry (Roche Applied Science) were applied for sequencing of the genomic DNA. The sequence reads were assembled with the GS Assembler Software (version 2.5.3.).

### Preparation and purification of 1,6-dihydroxyphenazine

*Brevibacterium iodinum* was cultured in 50 ml of DSMZ medium 1 for 4 days at 30°C and 200 rpm. 20 ml of this culture were used to inoculate 1.25 l of production medium consisting of 1% yeast extract, 1% glucose, 0.1% L-valine, and 1% Diaion HP-20 (pH 7.0 before sterilization). The production medium was cultured for 8 days at 26°C and 200 rpm, and the culture broth was extracted with dichloromethane (1.25 l). After filtration through a filter paper the crude iodinin extract was dried over Na_2_SO_4_ and evaporated to dryness resulting in 250 mg crude extract. Reduction of iodinin to 1,6-dihydroxyphenazine was carried out as previously described [20]. The obtained crude 1,6-dihydroxyphenazine (220 mg) was purified by chromatography over a silica gel 60 column using toluene/methanol (975∶25) as mobile phase. Fractions containing 1,6-dihydroxyphenazine were collected, pooled, and evaporated to dryness (yield 31.6 mg).

### Overexpression of Mpz10 protein and isolation of the membrane fraction

*mpz10* was amplified from genomic DNA of *Streptomyces* sp. SpC080624SC-11 using the primers mpz10_FW3 (5′-GAG ACG CCA TGG AAG AGC AAG GC-3′) and mpz10_RV3 (5′-GGT GCG AAT TCG CTC AGT TCT GG-3′). Underlined letters represent *Nco*I and *Eco*RI restriction sides, respectively. The amplified DNA fragment was digested with *Nco*I and *Eco*RI and ligated into the expression vector pET-28a(+) (Novagen), digested with the same restriction enzymes. The resulting plasmid pPH23 was verified by sequencing and restriction mapping.

From an overnight culture of *E. coli* Rosetta2(DE3)pLysS (Novagen) cells harboring pPH23 in Luria-Bertani medium with 50 µg ml^−1^ kanamycin and 25 µg ml^−1^ chloramphenicol, 17.5 ml were used to inoculate 500 ml of liquid Terrific broth medium [50] containing 50 µg ml^−1^ kanamycin and 25 µg ml^−1^ chloramphenicol. The culture was grown at 37°C and 250 rpm to an OD_600_ of 0.6. The temperature was lowered to 20°C, and isopropyl β-D-1-thiogalactopyranoside (IPTG) was added to a final concentration of 0.5 mM. The cells were cultured for further 14–15 h at 20°C and harvested by centrifugation at 6,080×*g* for 10 min. The resulting pellet was resuspended in Tris-HCl (50 mM, pH 7.5) and centrifuged again. 2 ml of lysis buffer (50 mM Tris-HCl (pH 7.5), 0.5 mg ml^−1^ lysozyme, 10 mM 1,4-dithiothreitol) were added per g cell weight and after stirring at 4°C for 15 min the cells were ruptured with a sonifier (Branson Sonifier W 250 D). To remove cell debris, the lysate was centrifuged at 5,000×*g* for 15 min. The resulting crude extract was passed through PD-10 columns (GE Healthcare) equilibrated with Tris-HCl (50 mM, pH 7.5) to gain a crude protein extract. The membrane fraction was isolated by centrifugation of the crude extract at 100,000×*g* for 75 min at 4°C. The obtained supernatant, containing all soluble proteins, was collected and the pellet was resuspended in Tris-HCl (50 mM, pH 7.5) and centrifuged for a second time at 100,000×*g* for 75 min at 4°C. Resuspension of the pellet in Tris-HCl (50 mM, pH 7.5) gave the membrane fraction. Protein concentrations were measured according to the method of Bradford [51].

### Assays for prenyltransferase activity

Reaction mixtures (100 µl) contained 50 mM Tris-HCl (pH 8.1), 10 mM MgCl_2_, 50 µg ml^−1^ membrane protein, 0.5 mM DMAPP, and 0.2 mM of 1,6-dihydroxyphenazine. Incubations were carried out at 30°C over 5 and 15 min. To examine substrate specificity the incubation mixtures (100 µl) contained 50 mM Tris-HCl (pH 8.1), 10 mM MgCl_2_, 1 mg ml^−1^ membrane protein, 1 mM of the aromatic substrate, and 1 mM of either DMAPP, geranyl diphosphate or farnesyl diphosphate. The mixtures were incubated for 2 h at 30°C. Incubations with membrane fraction from *E. coli* harboring the empty vector pET-28a(+) were used as negative controls. 5,10-dihydrophenazine-1-carboxylate was prepared as previously described [6] and was oxidized to phenazine-1-carboxylate with 15 µl of 100 mM sodium persulfate (Sigma) prior to extraction of the assay. Reaction mixtures were extracted with 100 µl of ethyl acetate/formic acid (975∶25). After vortexing and centrifugation, 70 µl of the organic layer were evaporated. The resulting residues were dissolved in 100 µl of methanol and 80 µl thereof were analyzed by HPLC using an Eclipse XDB-C18 column (4.6×150 mm, 5 µm; Agilent Technologies). Chromatography was carried out using a linear gradient from 40 to 100% solvent B in 12 min and additional 5 min at 100% solvent B (solvent A: water/formic acid (999∶1), solvent B: methanol/formic acid (999∶1)) at a flow rate of 1 ml min^−1^. For quantitative analysis of the product formation the absorbance was measured at 270 nm (1-hydroxyphenazine) and 275 nm (1,6-dihydroxyphenazine).

### Calculation of kinetic constants

For the calculation of *K*_m_ and *V*_max_ values GraphPad Prism software, version 5.01 for Windows (GraphPad Software Inc., La Jolla, USA) was used.

### Reduction of JBIR-46 to 1,6-dihydroxy-4-dimethylallyl-phenazine (DHDMP)

An authentic reference sample of JBIR-46 was dissolved in 200 µl of methanol. Subsequently, 4.8 ml of an aqueous Na_2_S_2_O_4_ solution (1 M) were added, and after vortexing, the mixture was extracted with 5 ml of *n*-hexane. The *n*-hexane layer was evaporated to dryness.

### Analysis by LC-MS

LC-MS analysis was performed using a Nucleosil 100 C18 column (100×2 mm, 3 µm; Dr. Maisch GmbH, Ammerbuch) at 40°C. A linear gradient from 40 to 100% solvent B in 12 min and additional 5 min at 100% solvent B (solvent A: water/formic acid (999∶1), solvent B: methanol/formic acid (999.4∶0.6)) with a flow rate of 0.4 ml min^-1^ was used. UV detection was carried out at 260, 275, 305, and 370 nm. Electrospray ionization (negative and positive ionization) in ultra scan mode with a capillary voltage of 3.5 kV and a capillary temperature of 350°C was used for MS analysis. MS/MS analysis was performed in positive ionization mode with a capillary voltage of 3.5 kV at 350°C. For MS/MS identification of the enzymatic products **1** and **2**, the masses 281 Da and 349 Da were selected for fragmentation, respectively.

### Production and purification of 3

The reaction mixture (25 ml) contained 50 mM Tris-HCl (pH 8.1), 10 mM MgCl_2_, 2 mg ml^−1^ membrane protein, 1 mM DMAPP, and 1 mM 1-hydroxyphenazine. After incubation over night at room temperature the mixture was extracted three times with 25 ml of ethyl acetate/formic acid (975∶25). The ethyl acetate layer was dried over Na_2_SO_4_ and evaporated. The dried residue was dissolved in 2 ml of methanol and purified by semipreparative reversed-phase HPLC using a Multospher 120 RP 18HP column (8×250 mm, 5 µm; Ziemer Chromatographie, Langerwehe) developed with 90% solvent B for 20 min (solvent A: water/formic acid (999∶1), solvent B: methanol/formic acid (999∶1)) at a flow rate of 2.5 ml min^−1^. Detection was carried out at 210 nm and the purification yielded 6.0 mg of **3**.

**1-hydroxy-4-dimethylallyl-phenazine (syn. 4-(3-methylbut-2-en-1-yl)phenazin-1-ol), 3**: Yellow powder; UV(MeOH) λ_max_ 239 nm (ε 2,780), λ_max_ 270 nm (ε 6,249), λ_max_ 367 nm (ε 887); IR (ATR) 3,360, 2,920, 2,851, 1,659, 1,632, 1,528, 762 cm^-1^; ^1^H and ^13^C NMR spectroscopic data see Table 3. HR-ESI-TOF-MS [M+H]^+^ *m/z* 265.1332 (calc. for C_17_H_17_N_2_O, 265.1335, Δ –1.1 ppm).

### Nucleotide accession numbers

The nucleotide sequences reported in this paper have been deposited in the GenBank database under the accession number KF808339.

## Supporting Information

Figure S1 **^13^C NMR spectra of 1-hydroxyphenazine (A, 101 MHz) and compound 3 (B, 151 MHz) in acetone-** ***d*** **_6_.** Additional resonances, observed in the ^13^C NMR spectrum of compound **3** are indicated by red numbers (B).
(TIF)

Figure S2 **^1^H NMR spectra of 1-hydroxyphenazine (A, 400 MHz) and compound 3 (B, 600 MHz) in acetone-** ***d_6_*** **.** Additional resonances, observed in the enzymatic product **3** are indicated by red numbers (B). Boxed regions show details of the coupling pattern of H-3 of 1-hydroxyphenazine (A) and of compound **3** (B).
(TIF)
