# Supplementary figures and images for: Genome-Based Discovery of a Novel Membrane-Bound 1,6-Dihydroxyphenazine Prenyltransferase from a Marine Actinomycete
Source: PLoS One. 2014 Jun 3;9(6):e99122. doi: 10.1371/journal.pone.0099122 (PMC4044012; doi:10.1371/journal.pone.0099122)

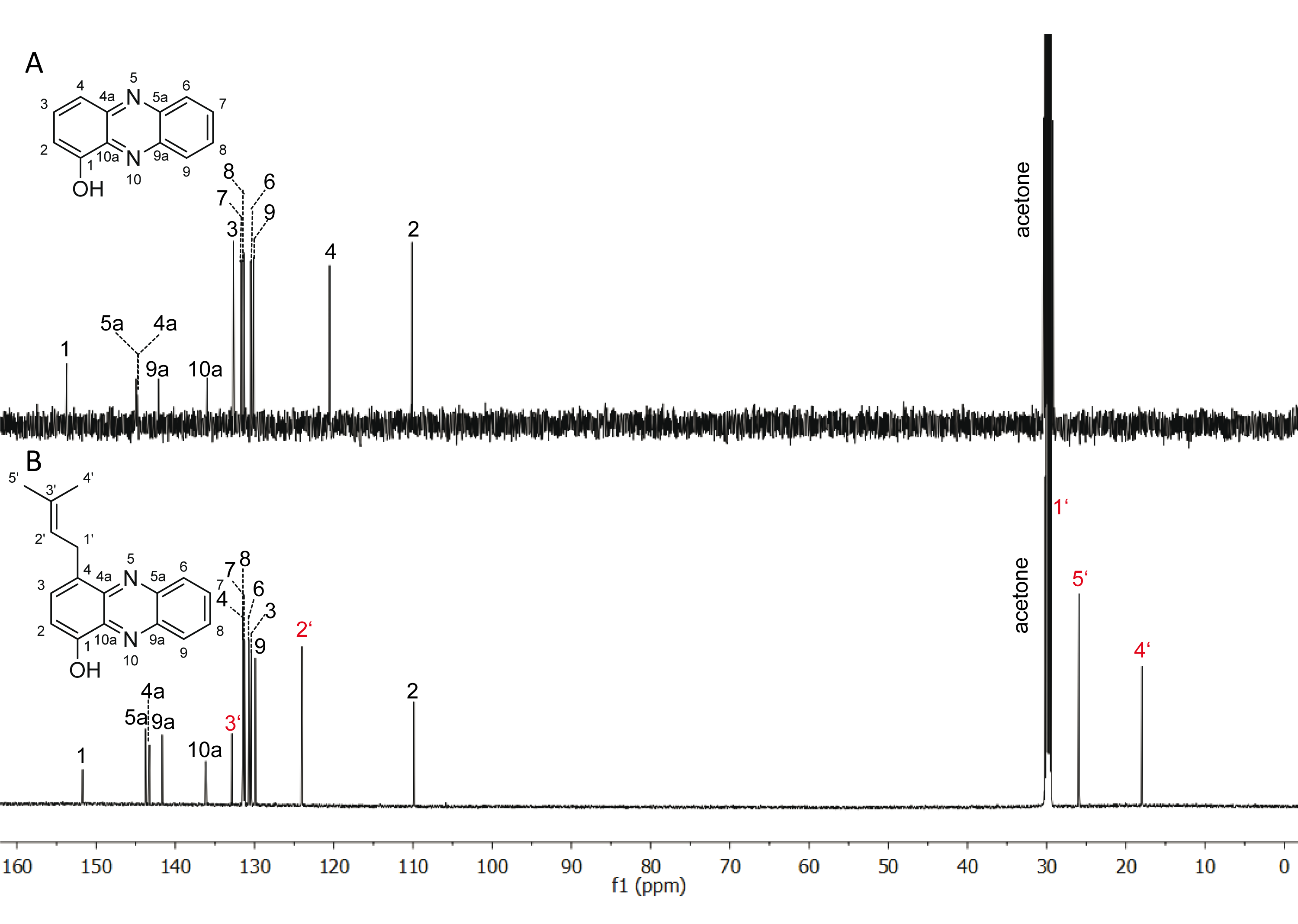

Supplement: Figure S1 — 13C NMR spectra of 1-hydroxyphenazine (A, 101 MHz) and compound 3 (B, 151 MHz) in acetone- d 6. Additional resonances, observed in the 13C NMR spectrum of compound 3 are indicated by red numbers (B). (TIF) [file pone.0099122.s001.tif]

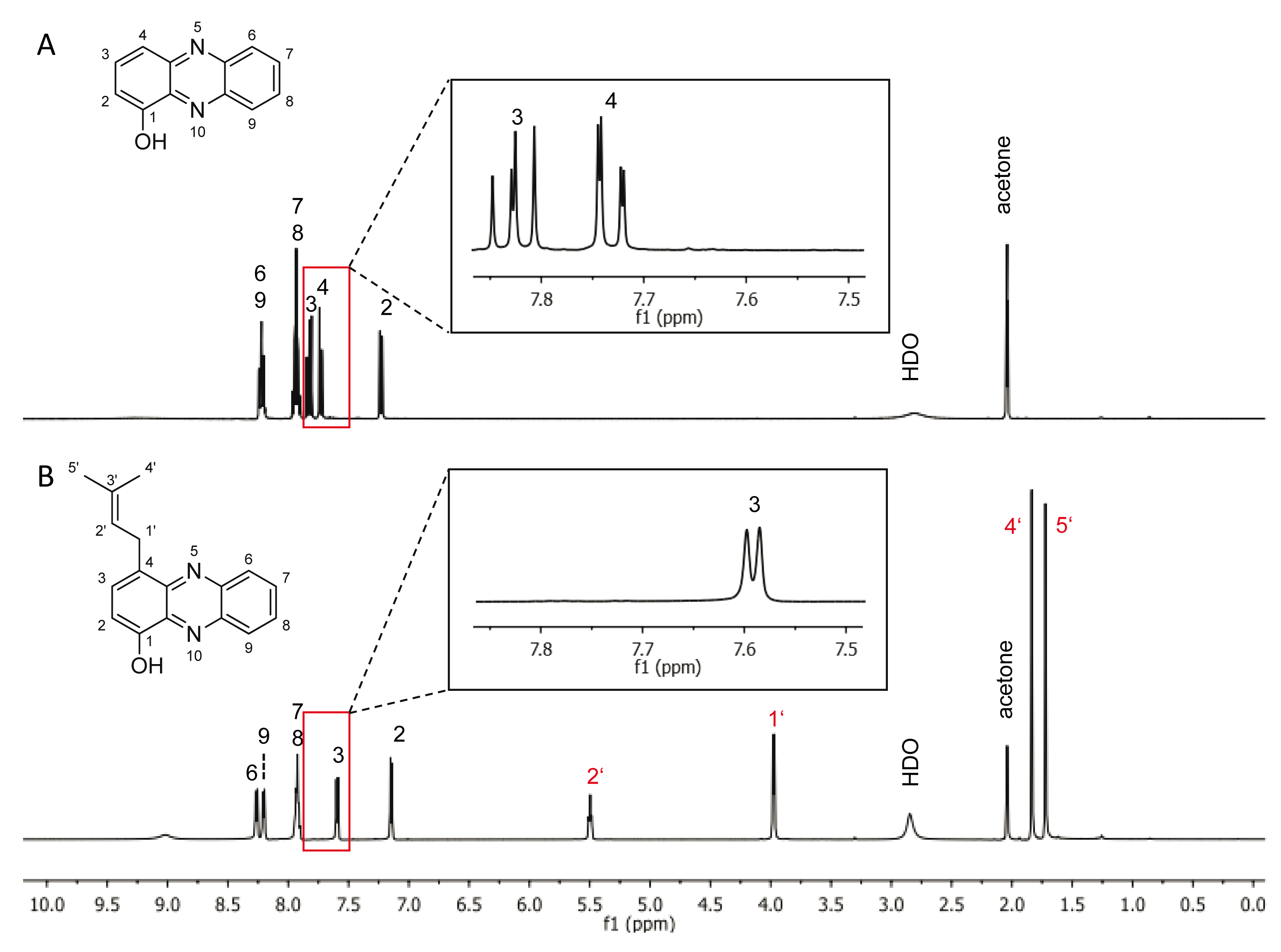

Supplement: Figure S2 — 1H NMR spectra of 1-hydroxyphenazine (A, 400 MHz) and compound 3 (B, 600 MHz) in acetone- d6 . Additional resonances, observed in the enzymatic product 3 are indicated by red numbers (B). Boxed regions show details of the coupling pattern of H-3 of 1-hydroxyphenazine (A) and of compound 3 (B). (TIF) [file pone.0099122.s002.tif]
